# Supplementary material for: Spray printing of organic semiconducting single crystals
Source: Nat Commun. 2016 Nov 22;7:13531. doi: 10.1038/ncomms13531 (PMC5121410; doi:10.1038/ncomms13531)
Supplement: Supplementary Information — Supplementary Figures 1-14, Supplementary Table 1, Supplementary Notes 1-6 and Supplementary References [file ncomms13531-s1.pdf]

Supplementary Information

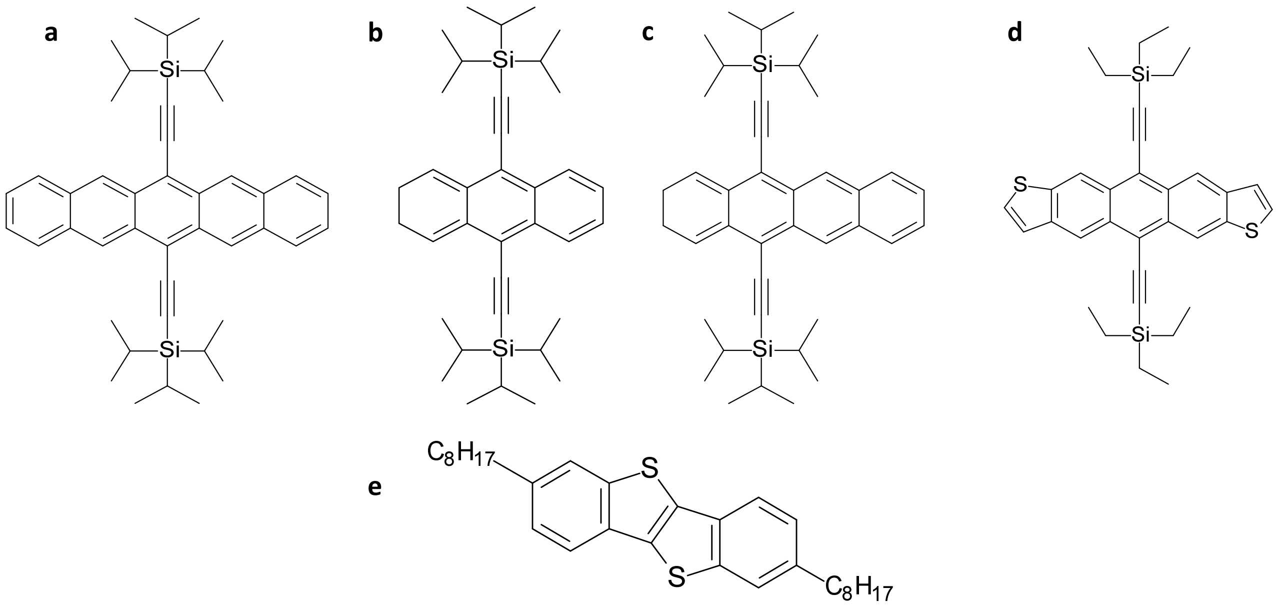

Supplementary Figure 1: *The chemical structures of solution processable semiconducting molecules. (a) TIPS-Pentacene, (b) TIPS-Anthracene, (c) TIPS-Tetracene, (d) TES-ADT and (e) C8-BTBT.*

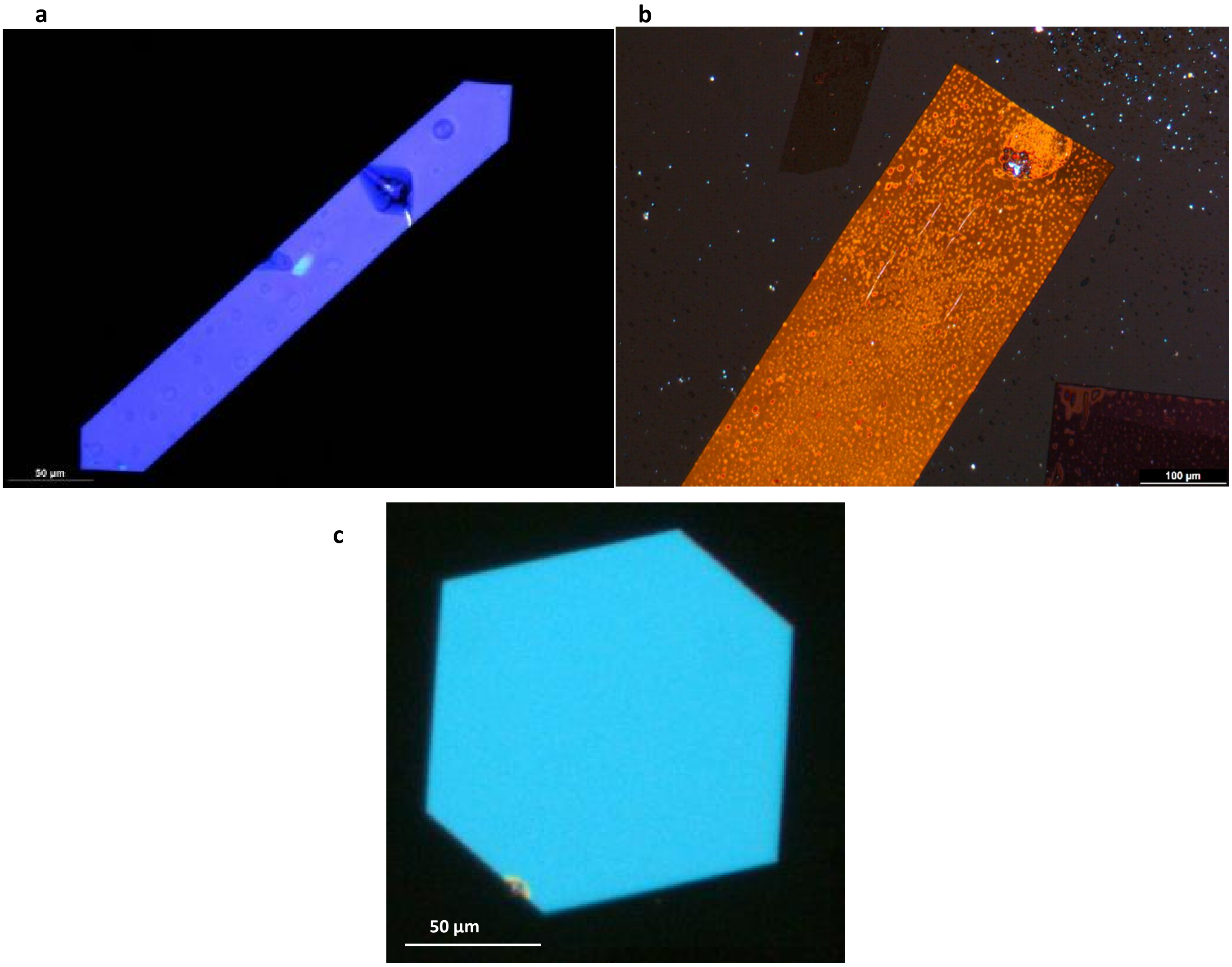

Supplementary Figure 2: *Cross polarised optical microscopy images of organic semiconductor single crystals. a) TIPS-Anthracene, b) TES-ADT and c) C8-BTBT.*

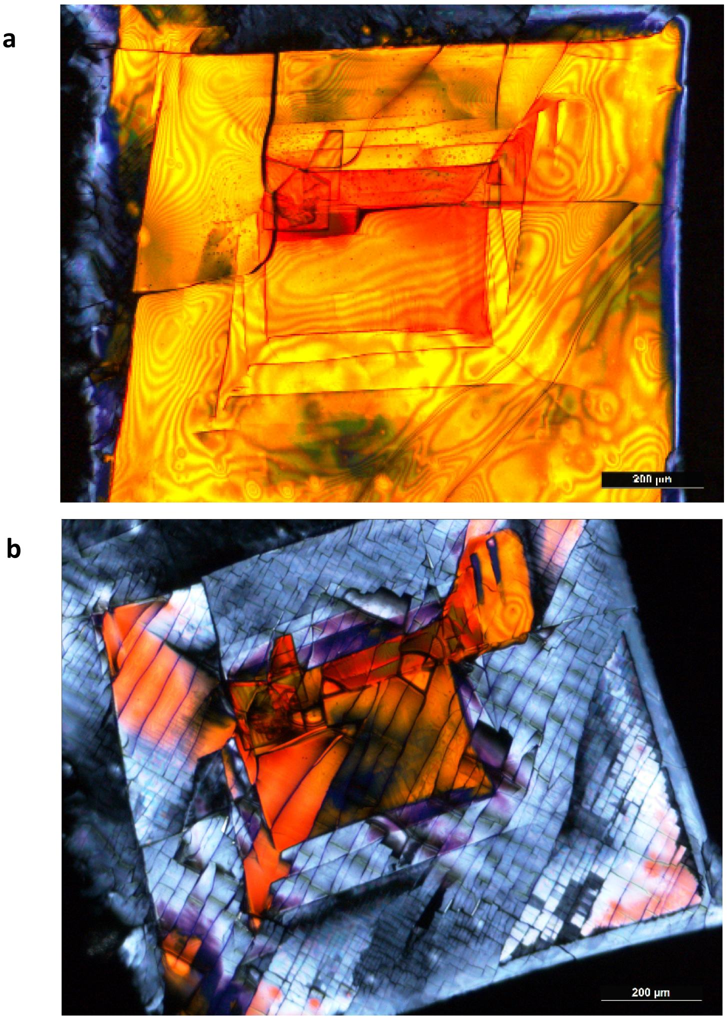

Supplementary Figure 3: *Cross polarised optical microscopy images of an OSSC of TIPS-Tetracene. (a) Image after 30 seconds and (b) Image after 60 seconds of light exposure. TIPS-Tetracene crystals experienced severe deterioration after exposure to light under the microscope illumination, exhibiting fracturing of the crystal.*

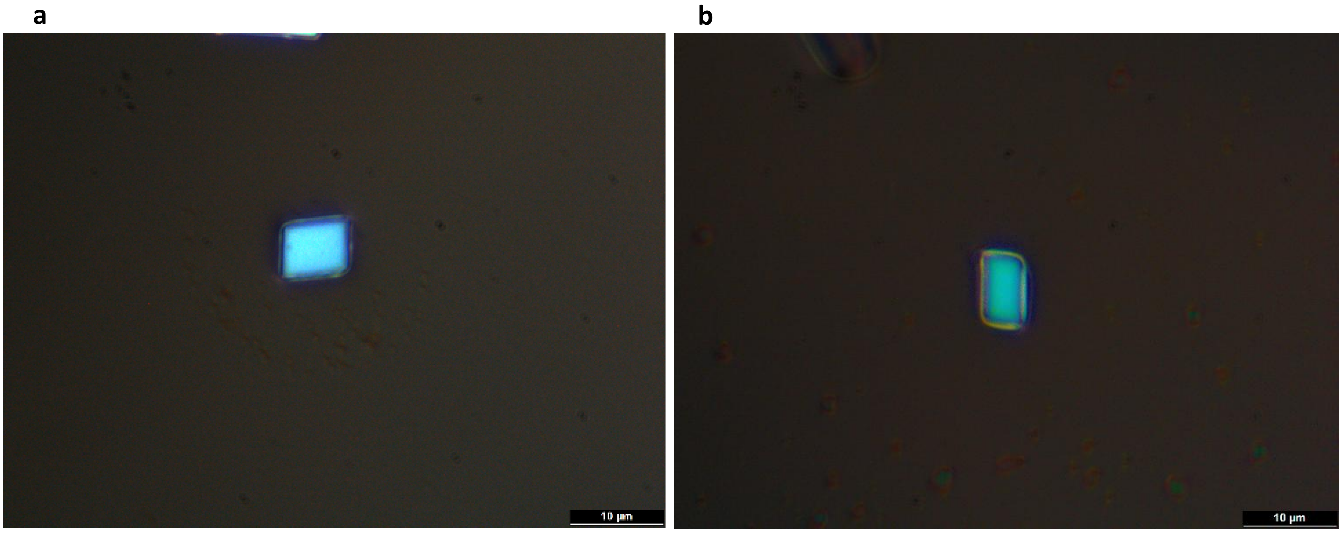

Supplementary Figure 4: *Cross polarised optical microscopy images of isolated TIPS-Pentacene OSSCs produced using spraying distances of 40 cm and various spraying angles. (a) Angle 15º and (b) Angle 75º .*

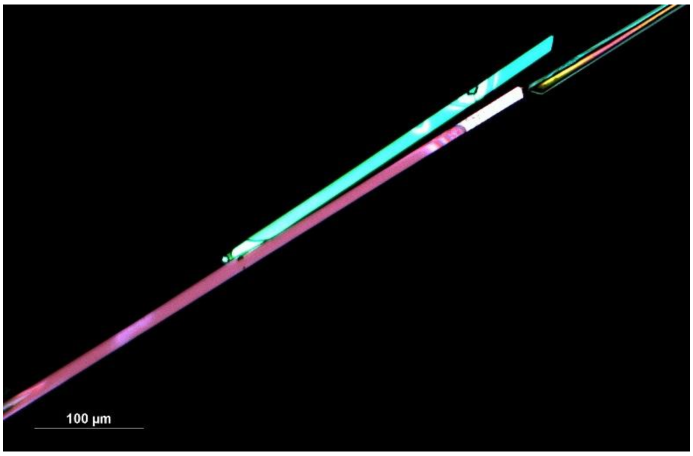

Supplementary Figure 5: *Polarised optical microscopy image of needle shaped crystals of TIPS-Anthracene, produced using a finer atomization airbrush.*

a **b**

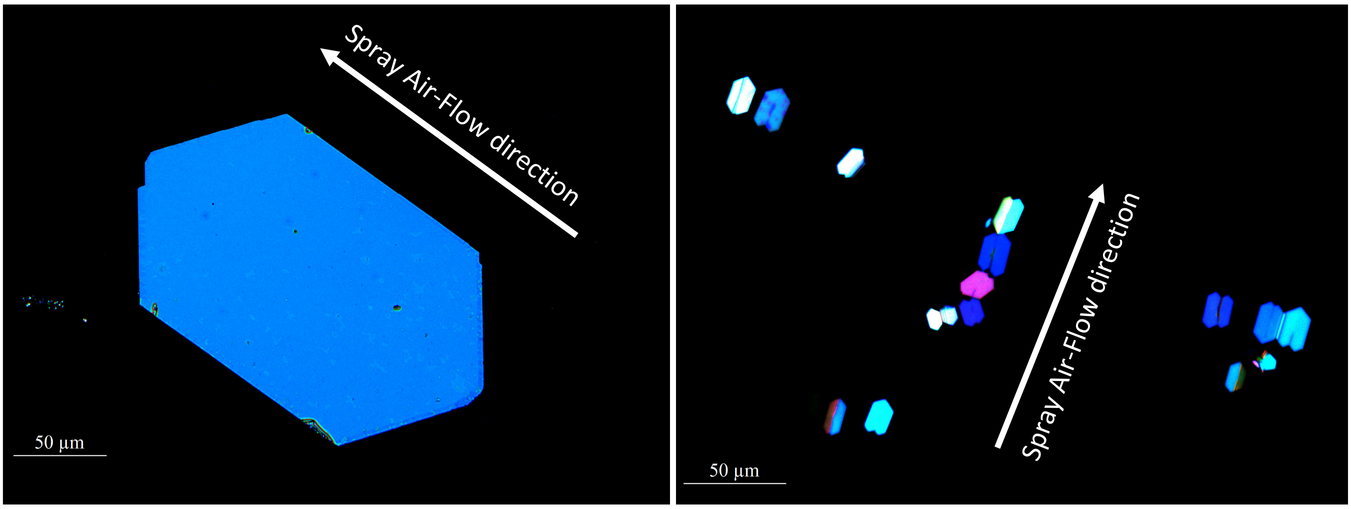

Supplementary Figure 6: *Polarised optical microscopy images of elongated C8-BTBT single crystals using various spray distances. (a) Distance 15cm and (b) Distance 30cm. The incident angle was set to 75° in both cases.*

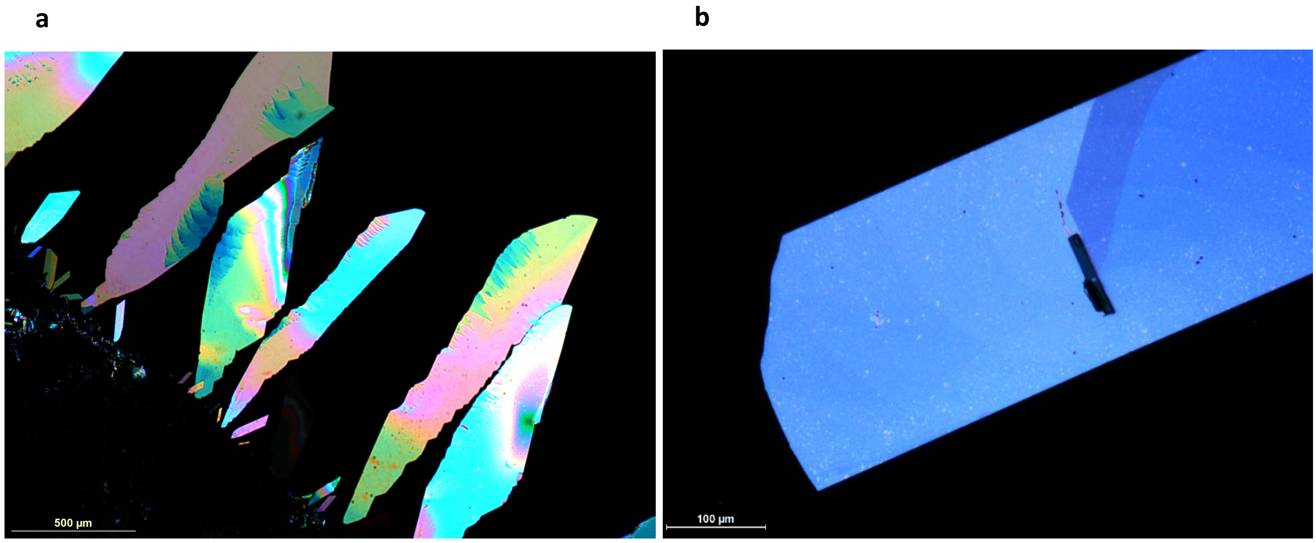

Supplementary Figure 7: *Polarised optical microscopy images of TIPS-Pentacene with different 'good' solvents. (a) Crystallites grown from tetralin solution, exhibiting non-uniformity in both the shape and thickness. (b) In contrast, uniform crystals were successfully patterned when toluene was used as the 'good' solvent of choice.*

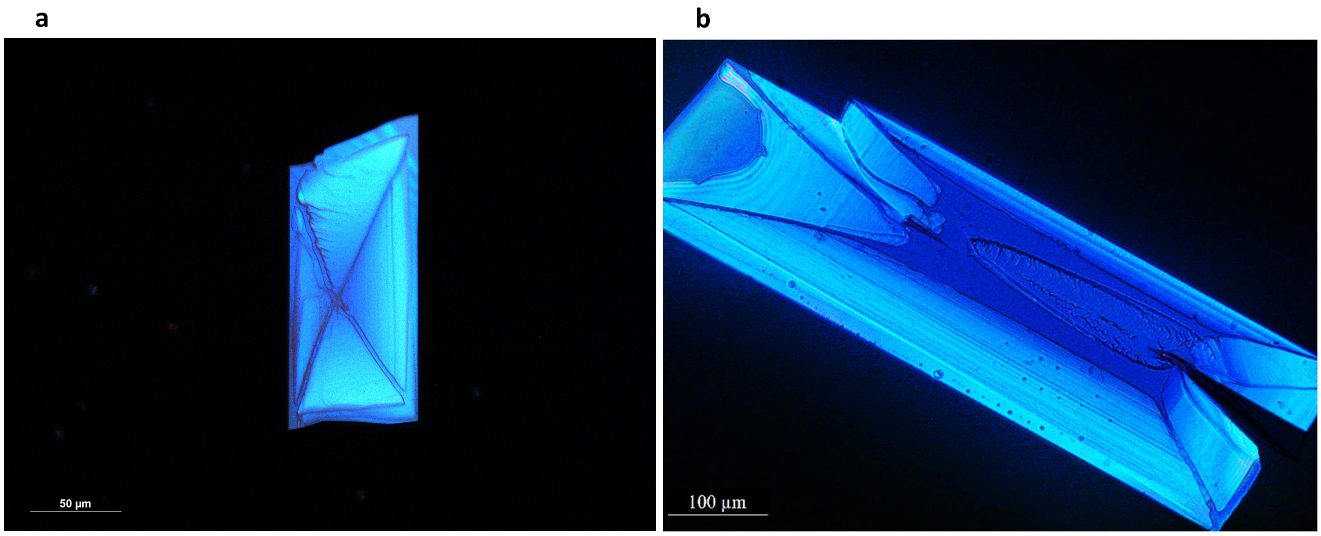

Supplementary Figure 8: *Polarised optical microscopy images of TIPS-Pentacene crystals grown using different antisolvents. (a) acetonitrile and (b) DMF as an antisolvent. Toluene was used as a 'good' solvent in both cases. Slower evaporating DMF resulted in larger crystals under the same printing conditions. Physical parameters for the solvents are given in Supplementary Table 1.*

a

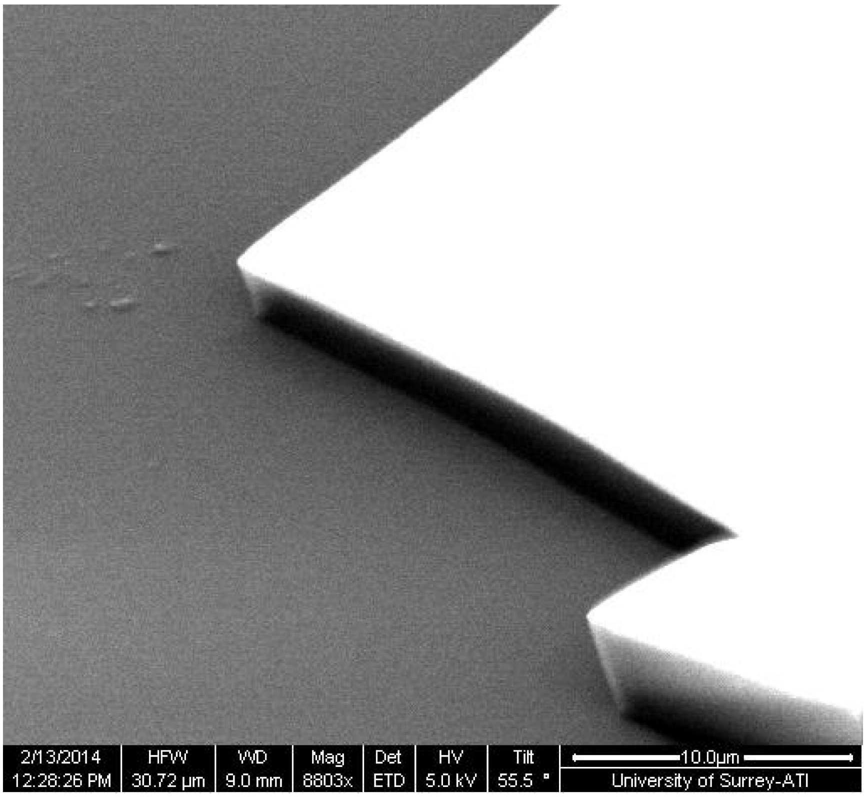

b

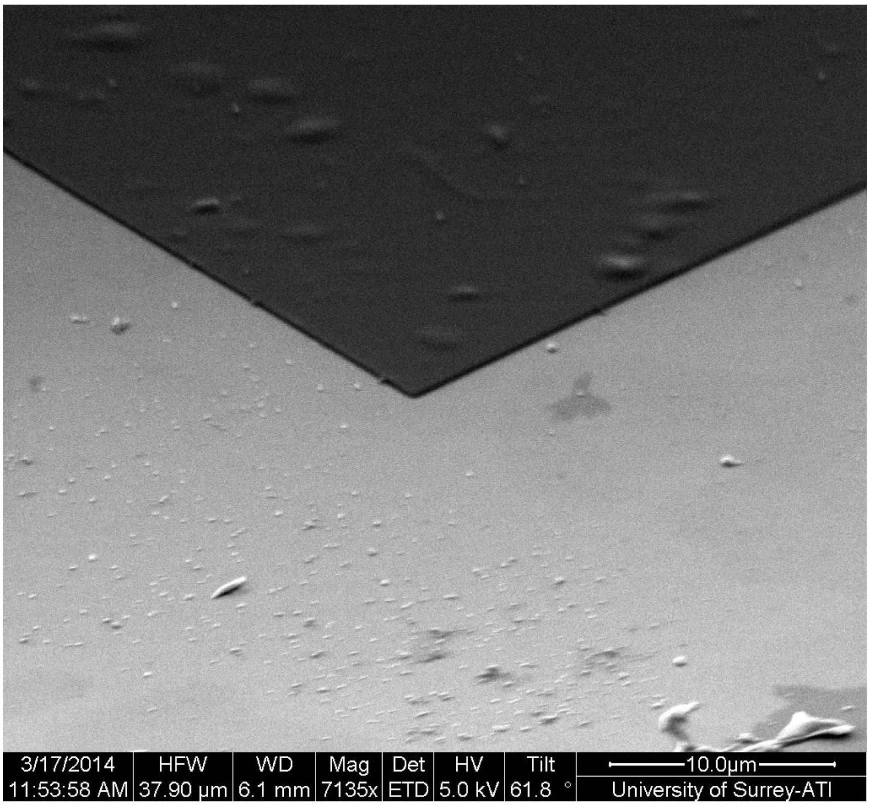

Supplementary Figure 9: *SEM micrographs of TIPS-Pentacene single crystals.* (*a) Crystals obtained from TIPS-PEN concentration in toluene 5mg/mL and (b) concentration 2mg/mL . Higher concentration resulted in thicker crystals.*

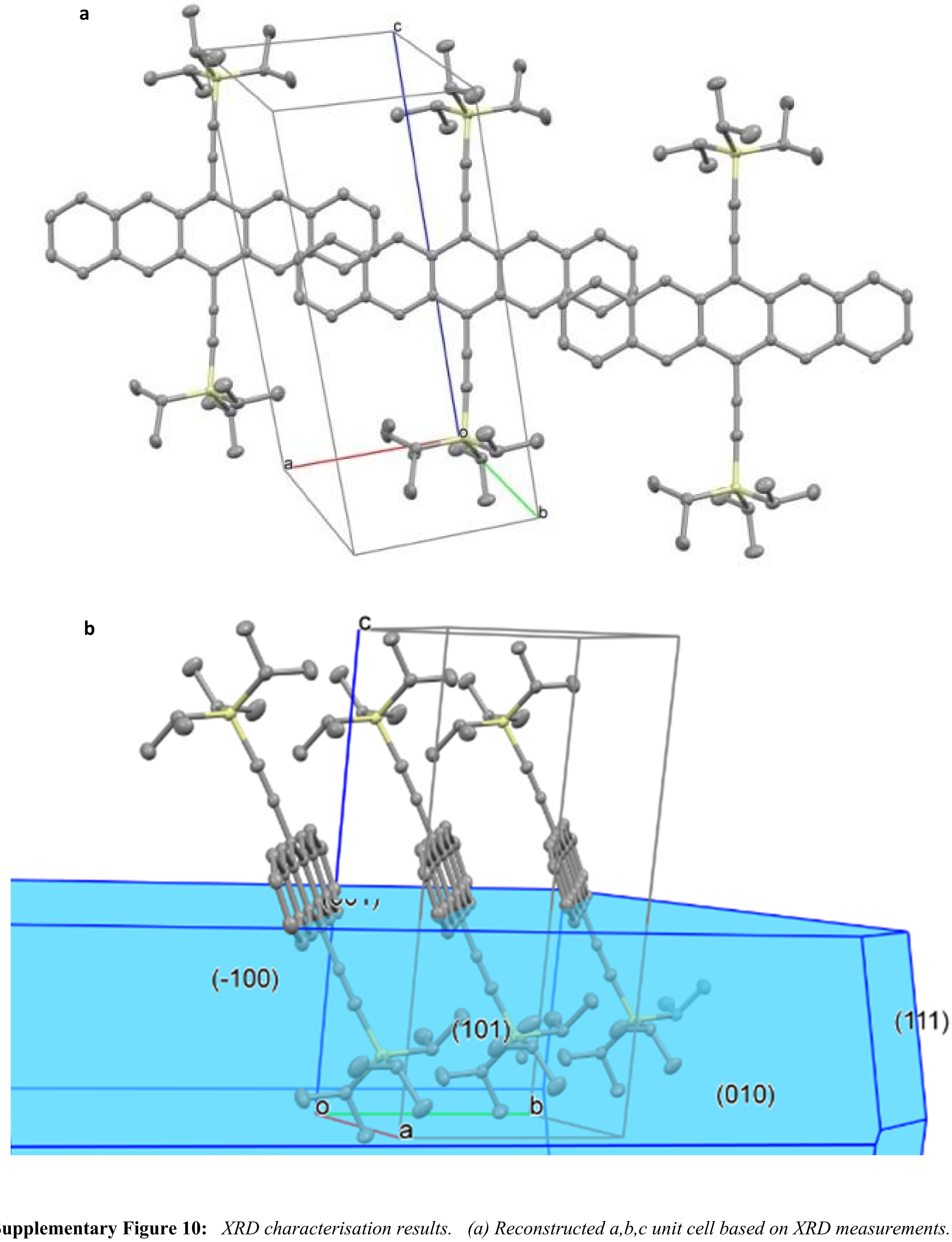

Supplementary Figure 10: *XRD characterisation results. (a) Reconstructed a,b,c unit cell based on XRD measurements. (a =7.562 Å, b=7.735 Å, c=16.844 Å, α = 89.57°, β = 78.50°, γ = 83.72°) (b) Unit cell orientation in respect to the crystal. Intermolecular π-π distances are 3.54Å.*

a

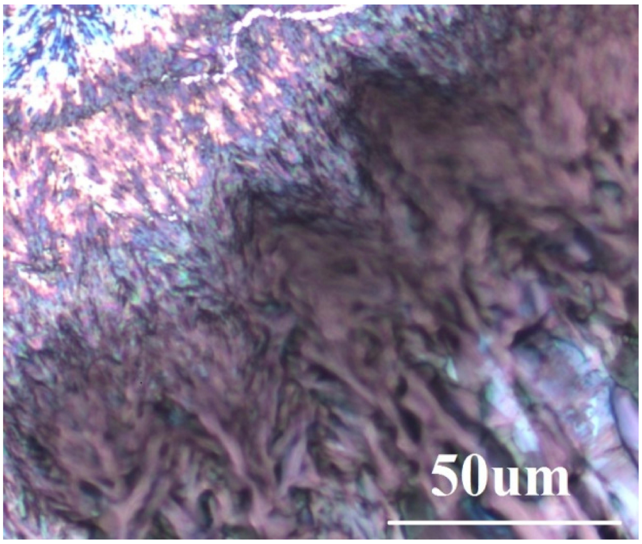

b

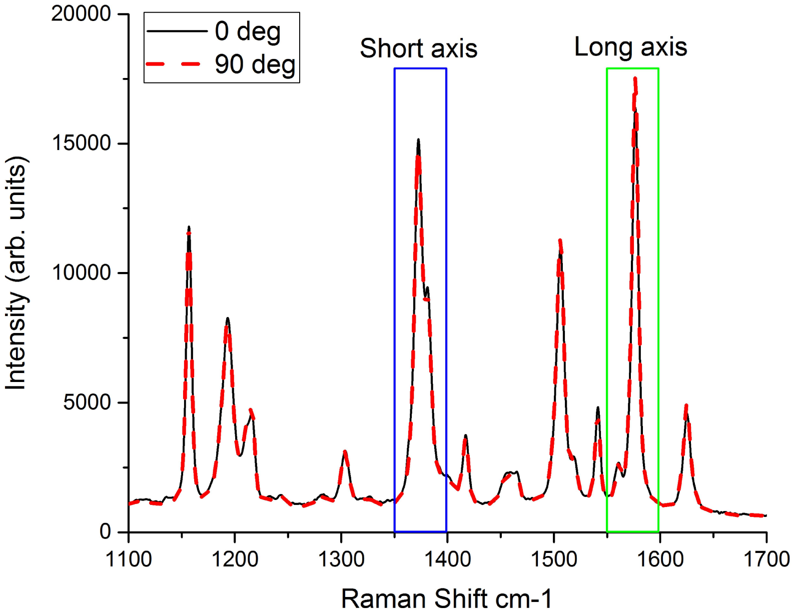

Supplementary Figure 11: *Highly poly-crystalline TIPS-PEN samples characterisation. (a) Optical microscope image of a highly polycrystalline sample that was examined, (b) p-Raman spectra of the same sample under two different polarization angles (0 and 90 degrees). The intensity of all the main modes remains unaltered despite the change of the angle.*

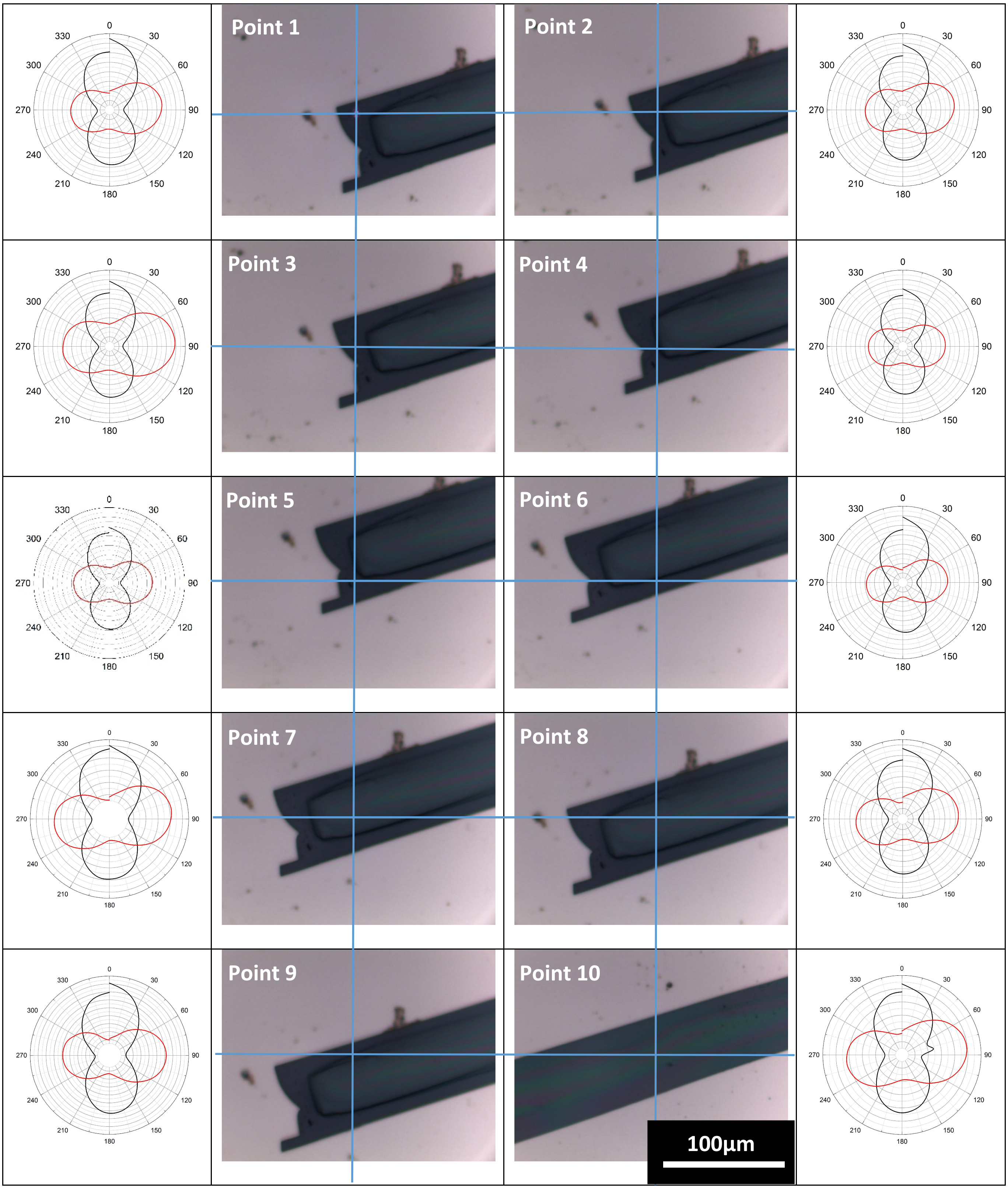

Supplementary Figure 12: *Optical images of the ten different points used to determine the molecular orientation uniformity along the crystal and the resulted p-Raman plots. The blue lines intersection represents the position of the measurement point. In all cases the data revealed consistent molecular orientation, which was independent of the examined location.*

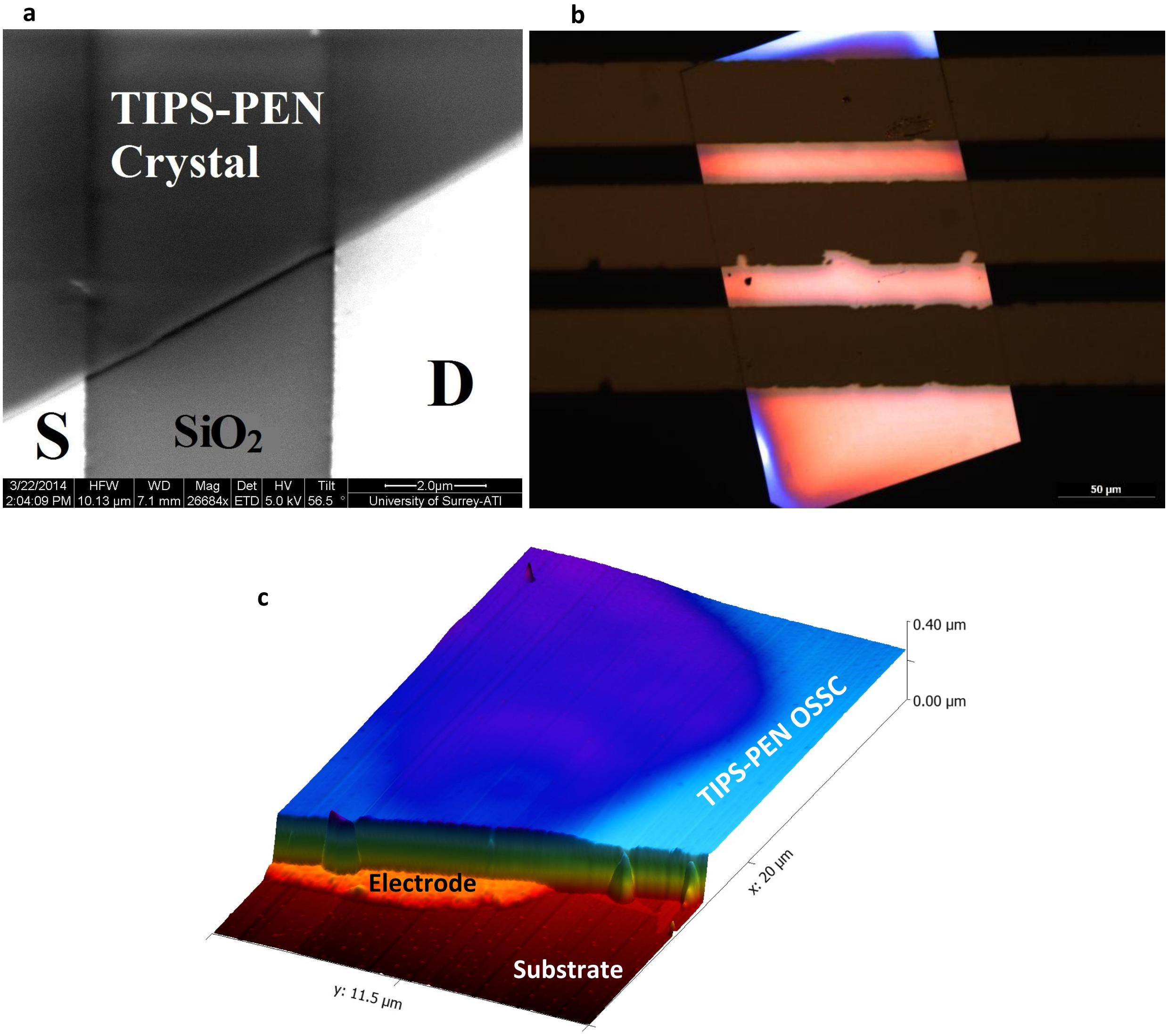

Supplementary Figure 13: *TIPS-PEN single crystal FETs. (a) BCBG FET architecture, (b) TCBG architecture. (c) AFM topography image of a single crystal deposited on top of a pre-patterned Au electrode. The crystal is following the morphology of the underlying substrate and electrode.*

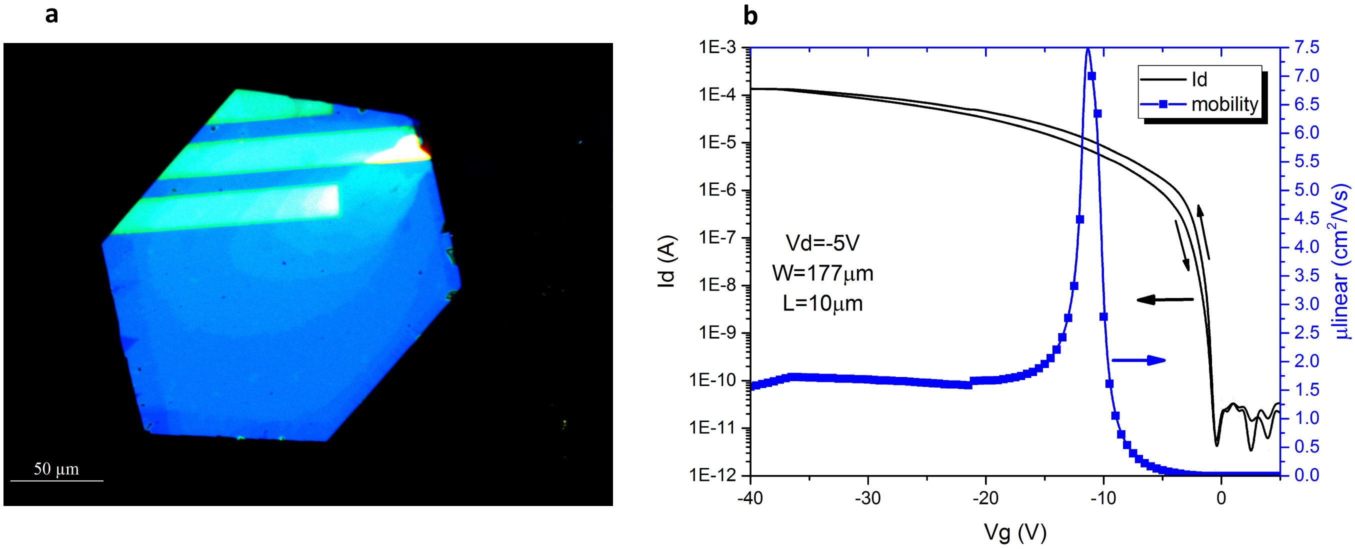

Supplementary Figure 14: *C8-BTBT single crystal FETs. (a) Polarised optical microscope image of a single crystal on top of pre-patterned electrodes, (b) FET transfer characteristics (black line) and mobility (blue line).*

Supplementary Table 1: Comparison of viscosity, surface tension and boiling point of the solvents used.

Good/Poor (G,P)	Solvent	Viscosity[1] (cP)	Surface Tension[1-3] (mN/m)	Boiling Point[1] (ºC)
G	Toluene	0.59	28.15	110.6
G	Tetralin	2.02	33.16	207.6
P	DMF	0.794	36.2	153
P	Acetonitrile	0.33	26.63	81.6

Supplementary Note 1: Crystal characterization

Characterization of single crystalline properties is typically based on a combination of techniques probing i) the entire crystalline structure uniformity, and ii) local molecular order.

i) The preferred characterisation of the whole crystal is polarised optical microscopy[4,5], allowing to inspect the appearance of crystal plates positioned between cross polarises, under a variety of magnifications typically ranging from x5 to x100. The smallest variations of crystal birefringence caused by crystal boundaries, crystallite orientations, defects and thickness changes are easily observable. Supplementary Fig. 3 provides a good example of easily identifiable crystal boundaries growth, seen as dark lines between uniformly coloured crystallites. Uniform, defect-free single crystals appear with highly consistent colour across the entire crystal area (e.g. Figs. 1d, 2a main text and Supplementary Figs 2c, 6a). Rotation of the crystal, such as TIPS-pentacene, in respect to the fixed crossed polariser positions varies the intensity of the crystal image, that extinguishes, when polariser axis coincides with optical axes of the crystal.

ii) Local molecular order it typically characterised by XRD, providing information on the crystal unit cell. (see main text and Supplementary Fig. 10). In addition to XRD, we have used polarised Raman spectroscopy which is also very sensitive to the local molecular orientations. A 2D polarised Raman scan thus provides a confirmation of identical molecular orientations within the studied crystal region (Supplementary Fig. 12)

Additional techniques, including SEM and AFM, complement the characterisation of single crystals, by providing information on crystals well defined edges, thickness, morphology (SEM); mono-molecular terraces, and top surface topology (AFM).

Supplementary Note 2: Crystals made with different semiconducting molecules

The proposed technique was applied for controlling the crystallization of TIPS-Anthracene, TIPS-Tetracene, TES-ADT and C8-BTBT. Results similar to those of TIPS-Pentacene single crystals were acquired (Supplementary Figures 2,3).

Supplementary Note 3: Spray printing apparatus parameters and crystal sizes

Larger spraying distances were consistent in producing smaller crystals by retaining the angle dependance in terms of their shape, such as square shape crystals shown in Supplementary Fig. 4a, or rectangular crystals in Supplementary Fig. 4b.

The number of crystals per area varies depending on the spray-printing parameters. Typically one crystal in an area of 350x260μm is grown when smaller spraying distances are used (Supplementary Fig. 6a). More than 10 crystals are typically grown in the same area, when larger spraying distances are used (Supplementary Fig. 6b). The crystals orientation is following the direction of the spray flow, thus allowing to control the desired orientation of the crystallites in respect to the substrate and forming reasonably well defined crystals arrays.

When an airbrush with finer atomization was used, needle shaped crystals were formed. Corresponding images are shown for TIPS-Pentacene in Fig. 1g (main text) and TIPS-Anthracene in Supplementary Fig. 5.

Supplementary Note 4: Different combinations of 'good' solvent and 'poor' solvent

Two 2mg/mL solutions of TIPS-Pentacene were prepared using toluene and tetralin as good solvents, respectively. The solutions were spray printed on top of several Si substrates covered with DMF (antisolvent) (see main text) and left to dry. Tetralin resulted in either polycrystalline patterns or non-uniform crystallites (Supplementary Fig. 7a). Toluene resulted in uniform crystals thickness, as described in the main text, with sharp and well defined boundaries (Supplementary Fig. 7b).

Supplementary Note 5: Highly poly-crystalline TIPS-PEN samples and p-Raman response

For verifying the ability of p-Raman to probe the crystallinity of an organic semiconductor, an isotropic sample was required. The technique developed for this purpose was Rapid Solvent Evaporation. A 5mg/mL solution of TIPS-Pentacene in toluene was drop-casted using a 0.5μL micro-pipette on a Si substrate, which was preheated at 90°C. Rapid evaporation of the solvent left limited time for the molecules to self-assemble into large crystalline domains, thus resulting a highly poly-crystalline film (Supplementary Fig. 11a), with a very large number of crystallites in the excitation laser area spot size (~8μm). Such highly polycrystalline sample can be effectively considered as an 'isotropic' sample due arbitrary orientation of the crystallites with the laser excitation zone. p-Raman spectra were acquired as described above. As Fig. 2e (main text) and Supplementary Fig. 11b show, the intensity of the dominant Raman peaks associated with the short and long axes of the molecule, remained unaltered indicating a highly disordered film.

Supplementary Note 6: Multiple points evaluation of the molecular orientation using p-Raman

Multiple 2D p-Raman scans on various locations over single TIPS-PEN crystal were performed. Several TIPS-PEN crystals were examined. The data (Supplementary Fig. 12) revealed consistent dependence between the intensity of the Raman signal and the polarisation angle of the monochromatic excitation light, indicating uniform stacking of the molecules inside the entire crystal structure.

Supplementary References:

1. Dean, J. A. *Lange's handbook of chemistry*. 15th edn, (McGraw-Hill, Inc., 1999).
2. Jasper , J. J. The Surface Tension of Pure Liquid Compounds. *J. Phys. Chem. Ref. Data,* 1, 841-1009, (1972).
3. Noda, Y. *et al.* Underlying Mechanism of Inkjet Printing of Uniform Organic Semiconductor Films Through Antisolvent Crystallization. *Adv. Funct. Mater.,* 25, 4022-4031, (2015).
4. Hartshorne, N. H. & Stuart, A. *Crystals and the Polarising Microscope*. 4th edn, (Edward Arnold Ltd., 1970).
5. Bloss, F. D. *Introduction to the Methods of Optical Crystallography* 1st edn, (Thomson Learning, 1961).
